# Part II of Finnish Agility Dog Survey: Agility-Related Injuries and Risk Factors for Injury in Competition-Level Agility Dogs

**DOI:** 10.3390/ani12030227

**Published:** 2022-01-18

**Authors:** Leena Inkilä, Heli K. Hyytiäinen, Anna Hielm-Björkman, Jouni Junnila, Anna Bergh, Anna Boström

**Affiliations:** 1 Department of Equine and Small Animal Medicine, Faculty of Veterinary Medicine, University of Helsinki, P.O. Box 57 (Viikintie 49), FI-00014 Helsinki, Finland; heli.hyytiainen@helsinki.fi (H.K.H.); anna.hielm-bjorkman@helsinki.fi (A.H.-B.); anna.bostrom@helsinki.fi (A.B.); 2 EstiMates Ltd., Kamreerintie 8, FI-02770 Espoo, Finland; jouni.junnila@estimates.fi; 3 Department of Clinical Sciences, Swedish University of Agricultural Sciences, P.O. Box 7054, 75007 Uppsala, Sweden; anna.bergh@slu.se

**Keywords:** dog agility, canine sports medicine, agility-related injury, sport-related injury, injury risk, agility training, risk factor, lumbosacral transitional vertebrae

## Abstract

**Simple Summary:**

Agility dogs can get injured during sports performance. Only a few factors have been linked to risk for injury. Through an online questionnaire, information was collected of more than 860 Finnish competition-level agility dogs. Of these dogs, 119 (14%) had suffered an injury in agility during calendar year 2019. Front limbs were most commonly injured. Typically, the injury presented as lameness. In general, dogs regained their performance level in four weeks, but 10% of injured dogs retired from the sport due to the injury. Dogs with multiple previous agility-related injuries or a diagnosis of lumbosacral transitional vertebra had higher odds of getting injured. Other common factors among the injured dogs included older age when starting course-like training and more than two agility-training sessions a week. A moderate number of monthly competition runs and an A-frame performance technique had lower odds of injury. This study identified new risk factors for injury in agility. This information can be used to improve the welfare of agility dogs.

**Abstract:**

Dog agility is associated with a risk for sport-related injuries, but few risk factors for injury are known. A retrospective online questionnaire was used to collect data on 864 Finnish competition-level agility dogs—including 119 dogs (14%) with agility-related injury during 2019. Data included injury details, health background, experience in agility, and sport and management routines prior to the injury. Risk factors for injury were evaluated with multivariate logistic regression. The rate of competition-related injuries was 1.44 injuries/1000 competition runs. The front limb was injured in 61% of dogs. In 65% of dogs, the injury presented as lameness. The main risk factors for agility-related injury during 2019 were multiple previous agility-related injuries (OR 11.36; 95% CI 6.10–21.13), older age when starting course-like training (OR 2.04 per one year increase; 95% CI 1.36–3.05), high training frequency, diagnosis of lumbosacral transitional vertebra, and physiotherapy every two to three months compared with never. The most important protective factors were moderate competition frequency and A-frame performance technique. These associations do not confirm causality. We identified new risk factors for injury in agility. This information can be used to improve the welfare of agility dogs.

## 1. Introduction

Agility is a growing dog sport with over 100,000 yearly competition runs in Finland alone [1]. Based on the four survey studies known to us, 8–33% of agility dogs suffer agility-related injury [2,3,4,5]. However, the evaluation periods of the four studies varied markedly, ranging from three months to potentially the whole sporting career of the dog [2,3,4,5]. The rate of agility-related injuries in competitions has been reported to be 2.12 per 1000 runs [3]. Almost half of the injuries in agility dogs require at least six to eight weeks for recovery, and 12% of injured dogs retire from agility due to the sport-related injury [2,4]. Most previous studies have been done almost exclusively in North American agility dogs [2,3,4,5]. However, regional differences occur in training and management routines [6,7], possibly affecting the risk of injury. Additionally, frequency of orthopedic conditions and injuries in agility dogs, including injuries not necessarily related to agility, also varies by geographic region [8]. Thus, occurrence of agility-related injuries and risk factor for injuries should be evaluated in different populations.

Some risk factors for agility-related injuries have been reported. Previous agility-related injury significantly increases the odds of additional injuries [7]. However, whether other musculoskeletal injuries and orthopedic conditions are associated with risk for agility-related injuries has, to our knowledge, not been evaluated yet. Chronic conditions could be provoked by agility or affect dogs’ ability to safely perform in the sport, predisposing to accidents.

Border Collie breed, the most common breed in agility, is consistently reported to be at increased risk of agility-related injuries [3,4,5,7,9], possibly due to their higher speed over obstacle sequences [10]. Speed of the dog may, however, be a risk factor independent of breed, but it has not been evaluated in earlier studies. In flyball, for instance, fast dogs were at increased risk of injuries [11]. Anecdotally, agility dogs perform at their full speed, and high velocities lead to increased kinetic energy. This may result in injury, for instance, in the case of collision with an obstacle, which is a common cause of agility-related injury [2].

In previous survey studies evaluating risk factors for injuries in agility, the competition, training, and management routines at the time of or prior to participation have been asked, without temporal association with injury [5,7]. However, these routines are not necessarily related to routines prior to the injury. This may be the reason why no relationship has been found between training or competition frequency and injuries [3,5,7]. Reverse causality may have caused musculoskeletal care, such as chiropractic care and massage, to be associated with agility-related injuries in previous studies, as these have been probably used for treatment of the injury [3,7].

Multiple other factors, such as field surface, fence height in relation to a dog’s height, and amount of daily exercise, have not yet been evaluated as risk factors for injury. The Finnish Agility Association acknowledged the lack of scientific knowledge regarding safety in agility and highlighted the need for research on this topic. Further information on obstacles, surfaces, or training routines associated with injuries could affect practices or regulations aiming to reduce injuries. More detailed knowledge on the dog-related factors associated with injury, such as size or history of musculoskeletal diseases or injuries, could improve identification of dogs at greater risk of injury.

The overall aim of this study was to provide a more complete understanding of agility-related injuries in competition-level agility dogs. The first specific objective was to describe agility-related injuries in Finnish competition-level agility dogs. The second objective was to provide information on training, competition, and management of agility dogs prior to the injury. Our last objective was to examine risk factors for agility-related injury. Based on previous studies and anecdotal evidence, we hypothesized that previous musculoskeletal injuries, increased training and competition frequency, and higher competition speed are associated with increased odds for agility-related injury during one calendar year, 2019.

## 2. Materials and Methods

### 2.1. Dogs and Respondents

Finnish owners and handlers whose competing agility dogs actively participated in agility and had had at least one agility-related injury during 2019 were included in this study. The dog had to have trained agility during 2019 and competed in Finland in 2018 and/or 2019. Injury was defined as an agility-caused clinical sign, evident within 24 h of sports performance, resulting in restriction of normal exercise and training. If the dog had suffered multiple injuries during 2019, only the latest one was described. The survey was completed by the owner or handler once per dog. If two surveys were sent in for the same dog, identified by the dog’s registration number, only the earlier answers were included. One respondent was allowed to complete the survey for multiple dogs. Data from non-injured dogs, collected by the same survey and published elsewhere [6], were compared with data from injured dogs in the risk factor analysis. The survey was distributed through the Finnish Agility Association and using social media (multiple Facebook pages). It was open to participation from July to September 2020.

### 2.2. Questionnaire

A Finnish language retrospective online survey consisting mainly of close-ended multiple-choice questions was developed using expert opinions, cognitive interviews, check lists, and a test group. The development of the questionnaire is described elsewhere [6]. The final survey (File S1) utilized skip logic, with only applicable questions shown to each respondent.

The questionnaire contained questions about signalment, the dog’s and the main handler’s experience in agility, and the health history of the dog. Moreover, questions dealt with the context of the injury, description of the injury, treatments used, and time to recovery (Table 1). Training practices and musculoskeletal care prior to the injury were also covered.

Background information was collected for both injured and non-injured dogs and consisted of variables such as age, size, highest competition level of the dog and handler, and previous diagnoses of musculoskeletal diseases, as earlier described in Part I of the study [6]. Training- and management-related items included for example frequency of training sessions, field surfaces, warm-up routine, musculoskeletal care, and exercise [6]. For dogs with an agility-related injury, questions covered training and management three months prior to the injury but were otherwise the same as in Part I of the study [6]. Musculoskeletal care was an exception to this, as practices one year prior to the injury were queried.

### 2.3. Competition Result Database

The Finnish Agility Association’s competition result database was used to retrieve information on each dog’s competition frequency during a three-month period prior to the injury. Additionally, the competition speed of the dog (mean speed of faultless runs) and the proportion of faultless runs were retrieved from the database and combined with the survey answers. Competition runs from both 2018 and 2019 were used to attain information on competition speed and proportion of faultless runs for as many dogs as possible.

### 2.4. Data Curation

In case of inconsistent answers, respondents were contacted by email. Answers were corrected according to email replies and open field descriptions.

Handling of the variables of age, competition years in agility, weight/height ratio, and health history was performed as previously described [6]. If the date of diagnosis of patellar luxation, osteochondritis dissecans (OCD), injury of the biceps tendon or muscle, injury of the supraspinatus muscle or tendon, shoulder instability/medial shoulder syndrome, fracture, other muscle injury, carpal sprain or sprain of a toe was not available, the information was considered missing. Only diagnoses made prior to 2019 were included to ensure these possible predisposing conditions had been present before the agility-related injury of 2019.

The anatomical locations of toe and nail were presented as a combined option in the survey but were handled separately using details from the open field descriptions and email replies. In cases where the respondent had selected almost all or all front limb locations, the exact location of the injury was considered ambiguous. Therefore, another anatomical location, “unspecified front limb site”, was added during data curation for these dogs.

For comparison of training-related routines of injured and non-injured dogs, some variables were categorized into fewer categories: Frequency of training sessions (<2, 2, or >2 sessions/week), training session length (up to 10 min, 10–15 min, at least 15 min) number of competition runs per month (<1.5, 1.5–<3.0, ≥3.0), main field surface during training and competitions (dirt/sand, artificial turf without filling, artificial turf with rubber filling, artificial turf with cork filling, or other), field surface during injury (as for main surface), and number of previous agility-related injuries (0, 1, or ≥2). To evaluate risk of collision with obstacle, the following three obstacle categories were created: jump obstacles (bar jump, spread jump, long jump, tire, and wall), contact obstacles (dogwalk, A-frame, and seesaw), and open tunnel and/or weave poles. Performance of each obstacle is shown in Video S1.

In non-injured dogs, training session frequency and length and main field surface were collected separately for winter and summer seasons [6]. To allow comparison with injured dogs, for non-injured dogs the answers of summer and winter season were combined, and only non-injured dogs with one categorized value for each of these variables (e.g., same frequency of training sessions during winter and summer seasons—or the dog had trained only during one season) were included in the risk factor analyses of each of these factors.

Sample size varied for each variable due to the skip logic and because “I don’t know” or “I can’t remember” answers were handled as missing values.

### 2.5. Data Analysis and Statistical Methods

A power analysis was utilized to calculate the sample size required to detect increased odds for injury during 2019, with a power of 80% and a confidence level of 95%, for the following parameters: (1) Mean competition speed of the dog (to detect a 0.2 m/s difference between injured and non-injured dogs, and (2) number of competition runs per month (to detect a difference of 0.5 runs per month). Data from previous studies were used to estimate injury rate [2,4]. To calculate means +/− SDs of speed and number of competition runs, results from the Finnish Agility Associations competition result database were used. The total number of required dogs (injured and non-injured summed) was 594 for competition speed and 251 for competition runs. However, all responses received during the data collection period were included and sample size was mainly dictated by available data.

Descriptive statistics (median and interquartile range for continuous variable, frequency tables for categorial variables) were calculated for all variables. Multivariate logistic regression analysis was used to evaluate whether an obstacle category and/or competition speed of the dog was associated with an obstacle collision. The same analysis approach was also used to investigate whether a field surface or obstacle category was associated with slipping during obstacle performance, and to investigate the association of field surface and competition speed with slipping elsewhere than during obstacle performance.

Potential risk factors for injury included all variables on background information, and training, competition, and exercise, with the exception of time off from agility, as reported in Part I of the study [6]. Time off from agility was not evaluated as a potential risk factor, as the values were not comparable due to differing time frames in injured (3 months) and non-injured dogs (one year). Training, competition, and management routines of injured dogs per year (musculoskeletal care) or per three months (all other variables) prior to their injury were compared with those of non-injured dogs during the year 2019.

Each factor was first evaluated using a univariate logistic regression, where each potential risk factor was assessed separately. Variables significant in univariate regression with *p* < 0.1 were included in the development of a multivariate model.

Second, to control for confounders, a penalized least absolute shrinkage and selection operator (LASSO) logistic regression model was fitted. LASSO is a regression analysis method that performs both variable selection and regularization to enhance the prediction accuracy and interpretability of the fitted statistical model. Akaike information criteria (AIC) was used as the criteria for optimal model selection, and Nesterov optimization as the optimization technique. The risk factors included in the optimal model were then used to fit a multivariate logistic regression model. Odds ratios (ORs) with 95% confidence intervals (CIs) were calculated from the model. Interactions between the risk factors were not evaluated.

Significance was set at *p* < 0.05. Statistical analyses were done using SPSS (version 26, IBM Corp., Armonk, NY, USA) and SAS (version 9.4, SAS Institute Inc., Cary, NC, USA).

## 3. Results

Continuous variables are presented as median (interquartile range). The total number of dogs varied between variables as a result of the skip logic and missing values.

### 3.1. Dogs and Respondents

**Figure 1:**
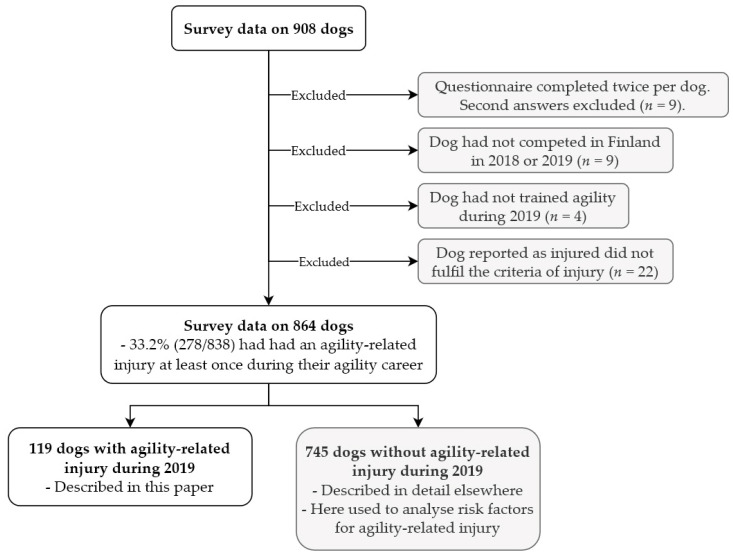
Dogs included in this study.

Survey data from 864 competition-level agility dogs were used to complete this study (Figure 1). The following paragraphs describe the agility-related injuries and injury preceding training, competition, and management routines of the 119 injured dogs provided by 117 respondents. Training, competition, and management routines of dogs without agility-related injury during 2019 (*n* = 745) have been described elsewhere [6], but they were used here to investigate which factors were associated with risk for agility-related injury during 2019.

The age of the dogs at the beginning of 2019 was 4.9 years (3.3–6.7 years). Weight and height were 15.0 kg (10.0–20.0 kg) and 48.0 cm (39.0–53.0 cm), respectively. Dogs represented the following height categories [12]: Extra Small (7.6%; height at withers <28 cm), Small (10.9%; 28 cm to <35 cm), Medium (20.2%; 35 cm to <43 cm), Small Large (26.1%, 43 cm to <50 cm), and Large (35.3%, ≥50 cm).

Of the sample of 119 dogs, 26.9% were intact females, 23.5% spayed females, 34.5% intact males, and 15.1% neutered males. Injuries were reported for dogs of 39 breeds. The most commonly injured breed was Border Collie (23.5%; 28/119). Table S1 provides the breeds of all injured dogs.

#### Dog’s and Main Handler’s Experience in Agility

The age when dogs had started course-like training (sequences of at least five obstacles) was 1.0 years (0.8–1.5 years, *n* = 115). Jumps were set at competition height at the age of 1.5 years (1.3–2.0 years, *n* = 116). Dogs had started competing in agility at the age of 2.3 years (1.8–3.0 years, *n* = 119). At the end of 2019, the length of the competition career was 3.1 years (1.6–5.0 years, *n* = 119). The dogs (*n* = 119) had the following highest competition levels from lowest to highest [6]: class 1 (20.2%), class 2 (16.8%), class 3 (31.1%), national championships (29.4%), and national team (2.5%).

The dogs’ main handlers had 10.0 years of experience in agility (6.0–15.0 years, *n* = 117). Almost half of the main handlers (43.6%; 51/117) had competed in national championships, or 3.4% (4/117) had been part of the national team. The highest competition level of the remaining handlers was class 1 (11.1%; 13/117), class 2 (11.1%; 13/117), or class 3 (30.8%; 36/117).

### 3.2. Context of the Injury

Most dogs (72.3%; 86/119) had had only one agility-related injury during 2019, with the number of agility-related injuries that year ranging between one and six for a single dog. The latest injury of 2019 will be described in the following sections.

#### 3.2.1. Training- and Competition-Related Injuries

About two-thirds (66.9%; 79/118) of the reported injuries occurred during training, while almost one-third (33.1%; 39/118) occurred during competition. Of the training-related injuries, 71.7% (33/46) occurred during the second half of the training session. When considering all runs during 2019 of the 864 dogs, the incidence of competition-related injury was 1.44/1000 competition runs. Completed competition runs during the same day prior to the competition-related injury ranged from zero to three (Table 2). In 62.3% (71/114) of the dogs, the injury was recognized only after agility, with the remaining cases (37.7%; 43/114) noticed during the agility session.

#### 3.2.2. Obstacle-Related Injuries

Most injuries occurred during obstacle performance (56.8%; 67/118). Some dogs (13.6%; 16/118) were moving between obstacles when injured, and in 4.2% (5/118) of the dogs the injury occurred in some other agility-related situation, such as at start line or during rewarding. The situation around the injury was unknown in 28.8% (34/118) of the dogs. Multiple options of injury-related factors had been chosen for four dogs. Table 3 shows the obstacles associated with injuries during obstacle performance. No injuries were associated with the flat tunnel or the wall jump.

Roughly a third (29.9%, 20/67) of the obstacle-related injuries resulted from a collision with an obstacle. Collision was associated with the three obstacle categories created (*p* = 0.001, *n* = 52); the odds of collision were decreased for contact obstacles (OR 0.03, 95% CI 0.00–0.30, *p* = 0.003), and open tunnel and/or weave poles (OR 0.05, 95% CI 0.01–0.43, *p* = 0.007) compared with jump obstacles. In the same regression model, competition speed was not associated with collisions (*p* = 0.547).

Of obstacle-related injuries, slipping during obstacle performance had occurred in 42.3% (22/52) of dogs. Obstacle category was associated with slipping during obstacle performance (*p* = 0.012, *n* = 46), with decreased odds of slipping during contact obstacles (OR 0.08, 95% CI 0.01–0.86, *p* = 0.037) compared with jump obstacles. Injury during open tunnels and/or weaves did not significantly differ from jump obstacles in the risk for slipping during obstacle performance (*p* = 0.143), and field surface was not associated with slipping in the same regression model (*p* = 0.517). Slipping elsewhere than during obstacle performance was reported in 16.7% (15/90) of dogs. Neither competition speed (*p* = 0.827) nor field surface (*p* = 0.323) was associated with slipping elsewhere than during obstacle performance.

Most dogs (72.7%; 8/11) with dogwalk-associated injury fell from the obstacle. Of dogs with an A-frame-associated injury, 40.0% (4/10) fell from the obstacle. Dogs with dogwalk-associated injuries performed the obstacle using the following techniques: stopped contact (60.0%; 6/10), running contact (30.0%; 3/10), or other or in between (10.0%; 1/10). All dogs (100.0%; 9/9) with an A-frame -associated injury used the running contact technique. Regarding performance technique, two dogs were excluded from the analysis because of inconsistent answers.

#### 3.2.3. Field Surface

Two-thirds (67.6%; 75/111) of the dogs injured themselves on a familiar surface on which they had trained or competed on a weekly basis during the three-month period prior to the injury. Table 4 shows field surfaces on which the injuries occurred.

### 3.3. Description of Injury

#### 3.3.1. Anatomical Location

The front limb was injured in 60.5% (72/119), neck or trunk in 34.5% (41/119), and the hind limb in 19.3% (23/119) of dogs. The most commonly injured anatomical locations are listed in Table 5. Additional injured anatomical locations included paw pad (front limb) (5.9%; 7/119), other location (5.9%; 7/119), digit (hind limb) (5.0%; 6/119), head (4.2%; 5/119), groin (4.2%; 5/119), metacarpal region (4.2%; 5/119), unspecified front limb site (3.4%; 4/119), stifle (3.4%; 4/119), elbow (2.5%; 3/119), antebrachium (2.5%; 3/119), and unknown (2.5%; 3/119). One injury (0.8%) was reported for each of the following: tail, hock, metatarsal region, crus, and nail of a hind limb. Multiple sites were reported for 37.8% (45/119) of the dogs.

#### 3.3.2. Type of Injury

Respondent-defined injury types in the order of incidence were muscle strain (42.0%; 50/119), unclear to respondent (18.5%; 22/119), ligament sprain (17.6%; 21/119), other (14.3%; 17/119), torn nail (10.1%; 12/119), abrasion (5.0%; 6/119), contusion (3.4%; 4/119), fracture (2.5%; 3/119), and laceration (1.7%; 2/119). Multiple types of injuries were reported for 11.0% (13/119) of dogs. Puncture wounds or dislocated joints were not reported.

#### 3.3.3. Clinical Signs

Lameness was the most common clinical sign (64.8%; 70/108), followed by pain on palpation or during passive range of motion assessment of joints (49.1%; 53/108), decreased weight bearing of a limb during standing (32.4%; 35/108), restricted range of motion in a limb and/or the trunk (30.6%; 33/108), stiff gait or stiffness when getting up (28.7%; 31/108), impaired performance (23.1%; 25/108), wound or bleeding (13.0%; 14/108), swelling (10.2%; 11/108), heat at injured area (10.2%; 11/108), or abnormal posture of a limb or trunk (5.6%; 6/108). Another clinical sign, such as restlessness or unwillingness to jump into the car, was present in 10.2% (11/108) of dogs. Multiple different clinical signs, ranging from two to seven, were reported in 77.8% (84/108) of dogs.

#### 3.3.4. Treatment and Recovery

Veterinary care was sought for 41.2% (49/119) of dogs due to their above-described injuries. The duration from injury to veterinary diagnosis was four days (one to 36 days). Therapies and paraprofessionals used in the treatment are listed in Table 6. Multiple treatment options were chosen in 76.3% (90/118) of the dogs. No treatment was given to 1.7% (2/118) of the dogs.

Recovery to normal daily exercise took 14 days (7–37 days, *n* = 114). Recovery to previous level in agility took 28 days (14–70 days, *n* = 88). Injury led to retirement from agility for 9.6% (11/114) of the dogs. Information regarding severity of injury was available for 99 dogs and is presented in Table 7.

### 3.4. Training, Competition, and Management Prior to Injury

#### 3.4.1. Agility Training

During the three months preceding the injury, the number of weekly training sessions ranged from less than one to seven. Most dogs trained one (35.6%; 42/118), two (41.5%; 49/118), or three (12.7%; 15/118) times per week. Typically, the active training time during one session was 5–10 min (18.6%; 22/118), 10–15 min (51.7%; 61/118), or 15–20 min (25.4%; 30/118). Weekly total training time was 18 min (13–25 min; *n* = 118) during weeks that the dog participated in agility. The usual relative jump height at training was 90% (77–98%; *n* = 118) of a dog’s height at withers.

The A-frame was performed using the following performance techniques: running contact (59.6%; 65/109), stopped contact (35.8%; 39/109), or other (4.6%; 5/109). The dogwalk was performed using the following performance techniques: stopped contact (53.6%; 59/110), running contact (40.0%; 44/110), or other (6.4%; 7/110).

#### 3.4.2. Competition

Dogs competed a median of three competition runs per month (0.7–5.0 runs per month; *n* = 119) during the three-month period prior to the injury. During 2018 and 2019 the competition speed of faultless runs was 4.6 m/s (4.0–4.9 m/s; *n* = 104), and the proportion of faultless runs was 16% (5–25%; *n* = 119). The maximum relative fence height in competitions was 103% (95–109%; *n* = 118) of the dog’s height at withers. Amount of weekly agility, combining training and competition, was 19 min (13–27 min; *n* = 112) during the weeks that the dog participated in agility.

#### 3.4.3. Field Surface

The main surfaces used in training and competition during the preceding three months included artificial turf with rubber (32.5%; 38/117), dirt or sand (26.5%; 31/117), artificial turf without filling (21.4%; 25/117), artificial turf with cork filling (12.0%; 14/117), artificial turf with sand filling (2.6%; 3/117), natural grass (1.7%; 2/117), fiber-sand mix (1.7%; 2/117), rubber mat (0.8%; 1/117), and horse-riding surface (0.8%; 1/117).

#### 3.4.4. Time off from Agility

During the three months preceding injury, 18.6% (22/118) of dogs had had time off from agility, with a total duration of 3.5 weeks (2.8–5.3 weeks; *n* = 18). The reasons for the break were planned break (e.g., periodization of training) (50.0%; 11/22), reason unrelated to the dog (27.2%; 6/22), previous injury or illness of the dog (22.7%; 5/22), or other dog-related reason (4.5%; 1/22). One dog had two reasons for a break.

#### 3.4.5. Warm-up and Cool-down Routines

Warm-up before agility was performed either always (95.8%; 113/118) or usually (4.2%; 5/118). The usual duration of the warm-up ranged from 5 min to more than half an hour—5–10 min (10.2%; 12/118), 10–15 min (28.0%; 33/118), 15–20 min (32.2%; 38/118), 20–25 min (16.1%; 19/118), 25–30 min (11.9%; 14/118), and over 30 min (1.7%; 2/118). Cool-down was performed always (88.1%; 104/118), usually (10.2%; 12/118), or sometimes (1.7%; 2/118). The usual duration of the cool-down ranged from less than 5 min to more than half an hour—below 5 min (0.8%; 1/118), 5–10 min (10.2%; 12/118), 10–15 min (22.9%; 27/118), 15–20 min (28.8%; 34/118), 20–25 min (16.1%; 19/118), 25–30 min (15.3%; 18/118), and over 30 min (5.9%; 7/118).

Table 8 shows the elements of a usual warm-up and cool-down. The respondents were able to select multiple items. The number of chosen items for the warm-up ranged from one to 11, with multiple items chosen for 96.6% (114/118) of dogs. For the cool-down, the number of chosen items ranged from zero to eight, with multiple choices reported for 90.0% (105/118) of the dogs.

#### 3.4.6. Musculoskeletal Care and Conditioning

The frequency of visits to professionals for musculoskeletal care is presented in Table 9. Most dogs (78.8%; 93/118) visited physiotherapist, massage therapist, osteopath. or other professional at least once every three months during the year preceding the injury. Conditioning exercises were performed by 78.8% (93/118) of dogs during the three-month period prior to injury. These exercises were done at least two times a week (18.3%; 17/93), once a week to every two weeks (44.1%; 41/93), or less often than every two weeks (37.6%; 35/93). Conditioning exercises were typically planned by the owner or handler (61.3%; 57/93), followed by the physiotherapist (30.1%; 28/93) and other persons (8.6%; 8/93).

#### 3.4.7. Daily Exercise

Total duration of usual daily walks was 1.5 h (1.3–2.0 h; *n* = 112) during the three-month period prior to the injury. During walks 4.2% (5/118) of dogs were always off leash, 46.6% (55/118) mostly off leash, 46.6% (55/118) mostly on leash, and 2.5% (3/118) always on leash. Besides agility, 12.7% (15/118) participated in other physically demanding activities such as canicross, herding, or hunting.

### 3.5. Health History

Most dogs (57.9%; 66/114) with agility-related injury during 2019 had suffered another agility-related injury prior to the latest injury of 2019. For 60 of these dogs, the number of previous agility-related injuries was known; the median of previous injuries was two (two to four injuries). Non-agility-related musculoskeletal injuries had occurred to 39.8% (45/113) of dogs during their lifetime.

Table 10 shows frequency of selected musculoskeletal diagnoses unrelated to the agility-related injury of 2019. Diagnoses of hip dysplasia, lumbosacral transitional vertebra or disease of the elbow were included regardless of the date of diagnosis, as these conditions are considered chronic. Other diseases were included as possible predisposing factors if they had been diagnosed before 2019. Diagnoses of iliopsoas injury, spondylosis, osteoarthritis, intervertebral disc disease, cranial cruciate tear, or luxation of the superficial digital flexor tendon were not reported by the respondents.

Of the 26 dogs with lumbosacral transitional vertebra, 76.9% had LTV1 (separation of first spinous process from the median crest of the sacrum or other mildly abnormal structure), 7.7% LTV2 (symmetrical LTV), 7.7% LTV3 (asymmetrical LTV), and 7.7% LTV4 (six or eight lumbar vertebrae).

### 3.6. Potential Risk Factors for Agility-Related Injury during 2019

Univariate regression analysis revealed 27 variables associated with increased or decreased odds of agility-related injury during 2019 with *p* < 0.1 (Table S2). These variables were selected for the development of a multivariate logistic regression model. Height category and previous agility-related injury (yes/no) were removed from the multivariate analysis since they were closely related to height and number of previous agility-related injuries, respectively.

Only dogs without missing data on any variables can be included in the development of the multivariate regression model. Due to the small sample size for the variables of frequency of training sessions, competition speed, and field surface variables, they were analyzed in separate subgroup models and were not included in the model for the full data. A small number of extreme (high) outliers were detected for three variables, and thus, the highest values were pooled so that these extreme outliers were not overemphasized in the statistical modelling results: weight (≥31 kg = 31 kg, *n* = 7), age at which course-like training was started (≥3.17 years = 3.17 years, *n* = 18), and age at which jumps were set at competition height (≥3.5 years = 3.5 years, *n* = 18).

**Figure 2:**
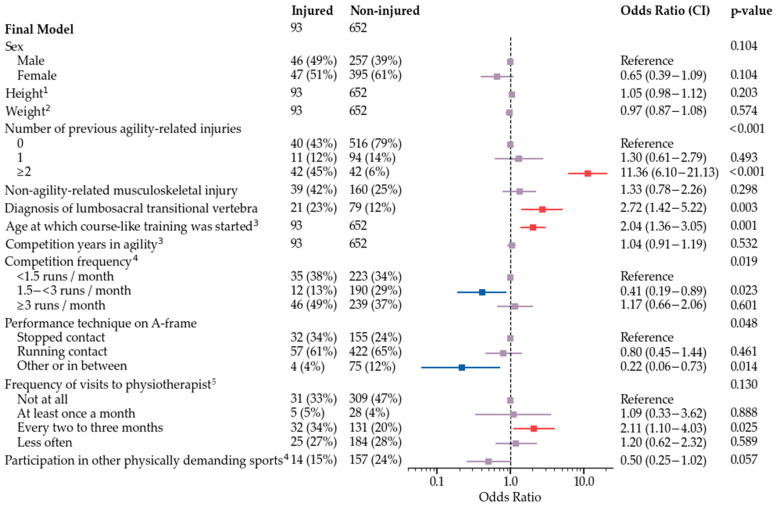
Training frequency model for odds of agility-related injury during 2019. ^1^ For a one centimeter increase, the odds increase by OR. ^2^ For a one year increase, the odds increase by OR. ^3^ For a one year increase, the odds increase by OR. ^4^ Routines during the three-month period preceding injury in injured dogs and during 2019 in non-injured dogs. ^5^ Routines during the one-year period preceding injury in injured dogs and during 2019 in non-injured dogs.

**Figure 3:**
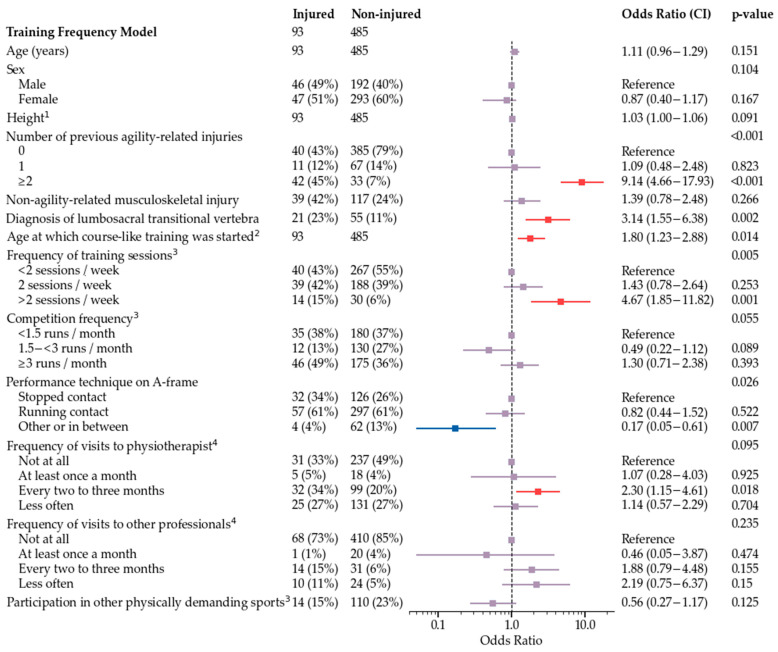
Training frequency model for odds of agility-related injury during 2019. ^1^ For a one centimeter increase, the odds increase by OR. ^2^ For a one year increase, the odds increase by OR. ^3^ Routines during the three-month period preceding injury in injured dogs and during 2019 in non-injured dogs. ^4^ Routines during the one-year period preceding injury in injured dogs and during 2019 in non-injured dogs.

**Figure 4:**
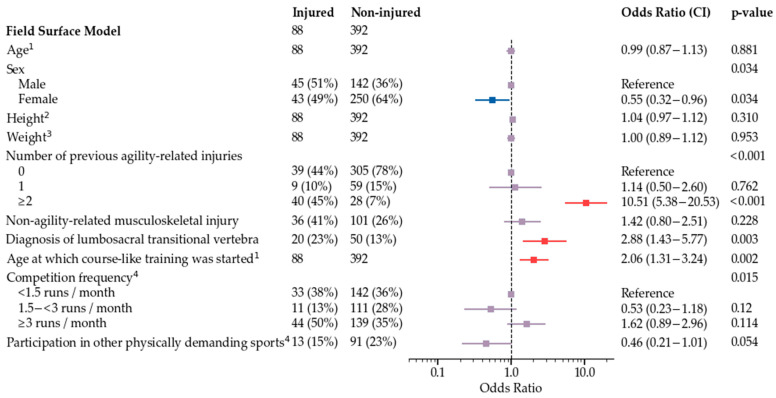
Field surface model for odds of agility-related injury during 2019. ^1^ For a one year increase, the odds increase by OR. ^2^ For a one centimeter increase, the odds increase by OR. ^3^ For a one kilogram increase, the odds increase by OR. ^4^ Routines during the three-month period preceding injury in injured dogs and during 2019 in non-injured dogs.

**Figure 5:**
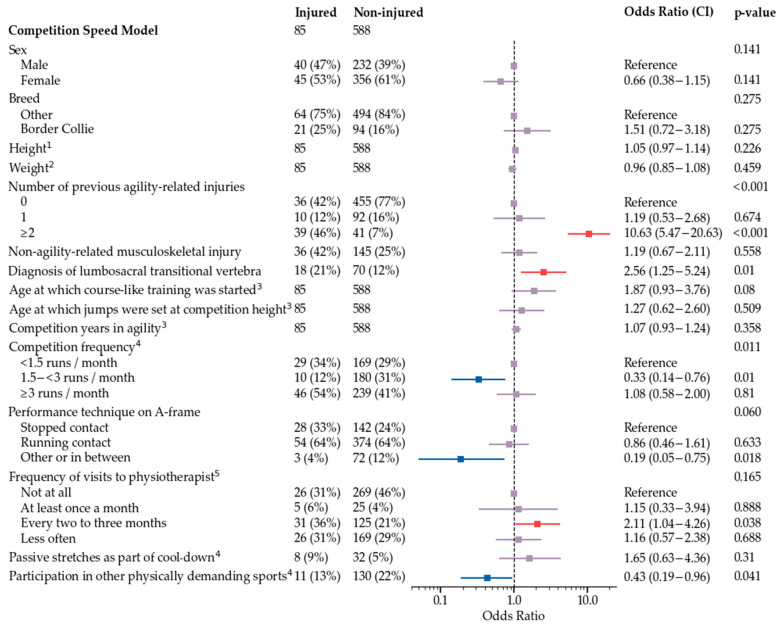
Competition speed model for odds of agility-related injury during 2019. ^1^ For a one centimeter increase, the odds increase by OR. ^2^ For a one kilogram increase, the odds increase by OR. ^3^ For a one year increase, the odds increase by OR. ^4^ Routines during the three-month period preceding injury in injured dogs and during 2019 in non-injured dogs. ^5^ Routines during the one-year period preceding injury in injured dogs and during 2019 in non-injured dogs.

The final multivariate model is shown in Figure 2. Subgroup models used all variables that were used for development of the final model, and additionally either frequency of training sessions (Figure 3), field surface at the time of injury and main field surface in training and competitions (Figure 4), or competition speed (Figure 5). Significant risk factors for agility-related injury during 2019 included multiple previous agility-related injuries, diagnosis of lumbosacral transitional vertebra, older age when starting course-like training, physiotherapy every two to three months compared with never, and more than two agility training sessions per week. Significant protective factors included moderate competition frequency, an A-frame performance technique other than stopped or running contact, and, in one model, participation in other physically demanding sports. Competition speed or field surface variables were not included in the speed model or the field surface model, respectively.

The anecdotal assumption being that starting training at a young age increases odds of injury, additional univariate logistic regression analysis was done to evaluate association between age at which course-like training was started and agility-related injury during the whole career (during 2019 or earlier). No association was observed (*p* = 0.919).

## 4. Discussion

This study provides information on agility-related injuries of competition-level Finnish agility dogs during 2019. Over 10% of the agility dogs in our study suffered an agility-related injury, with most injuries occurring in training and presenting as lameness. The rate of competition-related injuries was 1.44 injuries/1000 competition runs. Front limbs were most prone to injuries. As hypothesized, previous agility-related injuries were a significant risk factor for agility-related injury during 2019. Information on non-agility-related musculoskeletal injuries improved the logistic regression models, but was a non-significant factor, and thus, its role remains unclear. Contrary to our hypothesis, competition speed of the dog did not contribute to the injury risk. As expected, high training frequency was associated with increased odds of injury.

### 4.1. Injury Rate

In our sample, almost a third of the dogs had suffered an agility-related injury during their career, which is in agreement with most previous studies [2,5]. Higher proportions of injured agility dogs, almost 50% of Scandinavian agility dogs and over 40% of agility dogs worldwide, have been reported recently [8]. However, that study included also non-agility-related injuries and orthopedic conditions, likely leading to the higher proportions than in other studies [8]. The incidence of sport- or work-related injuries appears lower in agility dogs than in greyhounds, working farm dogs, or gundogs [13,14,15]. In flyball dogs, injury risk appears to be similar to agility dogs [11,16]. However, the definition and recording of injuries varies across the studies, making comparisons difficult. In our sample, the rate of competition-related injuries was slightly lower than the previously reported rate of 1.72 injuries/1000 runs in North American agility dogs [3]. Course designs and regulations are likely to differ between Finland and North America, possibly leading to different injury rates. Additionally, management and training routines in Finland differ from those in the USA [3,6,7], which could affect injury rate in competitions.

Most injuries in our study occurred during training, with most training-related injuries occurring in the second half of the training session, suggesting that fatigue may be involved. Similarly, three-quarters of competition-related injuries occurred in the second or later run of the day, when anecdotally dogs complete two to three runs per event. High-impact activities should be avoided when the dog is fatigued to prevent injuries [17]. Thus, handlers and coaches should be educated in detecting signs of fatigue such as excessive panting, muscle trembling, and gait changes [18]. However, the usual length of a training session as such was not associated with increased odds of injury in our sample.

### 4.2. Obstacle-Related Injuries

The obstacles most commonly involved in injures, according to earlier reports, are bar jump, A-frame, and dogwalk [2,4]. Interestingly, in addition to these obstacles, injuries during performance of open tunnels were relatively common in our study, whereas previously open tunnels have only seldom been associated with injuries [2,4]. Observations from the field suggest that the speed of the dogs has increased over the years, materials of tunnels have evolved to improve the grip for the dog, and the attachments of tunnels has become more fixed—possibly increasing the hazards of open tunnels. A larger sample of tunnel-related injuries will be needed to determine which tunnel-, dog-, or course design-related factors are associated with injuries in tunnels.

Traumatic injuries resulting from normal sport activity or sport-related accident are considered rare in most canine sports [17]. However, contradictory to this assumption, collision with an obstacle was a relatively common cause of agility-related injury in our sample as well as in previous studies [2,4]. In our study, collisions were particularly common on jump obstacles compared with other obstacles, possibly because they are the most common obstacle type on the course [2]. Attention should be paid to materials of obstacles; lightweight obstacles are going to reduce the impact to the dog in the case of collision with the obstacle. Multiple potential causes, such as handling error, course planning, slippery surface, fatigue of the dog, or dog’s visual impairment, may lead to a collision. Analysis of video material available from collisions might help to identify some of the most common causes.

Falls from A-frame and dogwalk have not been separated from collisions in previous studies, although both obstacles have been commonly associated with injuries [2]. In our sample, a fall was common among dogwalk-associated injuries. Equipment recommendations and rule changes have been suggested as possible aids to reduce sport-related injuries in human sports [19]. Thus, falls from the dogwalk could probably be reduced by changing the regulations for the obstacle’s dimensions, with width currently being only 30 cm [20]. In addition to obstacle dimensions, an angled approach at speed may be a factor leading to falls from the narrow plank. Regulations should be updated regularly to ensure safe performance, also at the higher speeds of modern agility dogs. Such changes have already been made regarding the tire obstacle, which must break in case of collision [20], possibly decreasing the proportion of injuries associated with the tire from the previously reported 6% to below 2% in our study [4]. Similar improvements should be made for the dogwalk and the A-frame, which are commonly involved in injuries despite being performed on a course much less frequently than bar jumps [2].

All dogs with A-frame-associated injury in our sample had been trained to perform the obstacle with the running contact technique, although in the whole sample also other techniques were common. This suggests that the running contact technique may be associated with a higher risk for A-frame-related injuries. A larger sample size is required to further assess injuries related to the A-frame. When evaluating risk for agility-related injury in general, the running contact technique used at the A-frame was associated with similar odds of injury as the stopped contact technique. Dogs with other, or in between, techniques had lower odds of agility-related injury. Anecdotally, these dogs generally decelerate on the descending part of the A-frame without an abrupt stop at the end. This may decrease stress on the musculoskeletal system and reduce the risk of injuries in general.

### 4.3. Anatomical Location and Treatment

In our study, six out of ten dogs injured their front limbs, which is a higher proportion than previously reported in agility dogs [5]. The reason for this difference is unknown. Front limbs are, however, subjected to high demands during obstacle performances. During jumping higher peak vertical forces are applied to the front limbs than to the hind limbs; peak vertical forces are on average 2.5 times the body weight for each front limb in advanced agility dogs [21]. Excessive carpal extension, outside the reported passive range of motion values, has been described at first contact with the A-frame and when landing from a jump [22,23,24,25]. Additionally, jumping and performing A-frame requires marked activation of front limb muscles in the shoulder region [26,27]. Thus, the high proportion of front limb injuries is not surprising. To take this into account, agility dogs could benefit from coordination, proprioception and strength training targeted especially to front limbs. Future studies should evaluate the effect on professionally-planned conditioning programs on injuries in agility.

Veterinary care was sought only for 40% of the injuries in our sample, whereas in previous reports, in mainly North American populations, the proportion has been 61–78% [2,4]. The recovery times suggest that minor injuries were slightly more common in our sample [2,4], which could explain some of this difference. Lack of proper diagnosis may lead to insufficient treatment and rehabilitation in some cases, potentially resulting in re-injury.

### 4.4. Risk Factors

Previous agility-related injuries have been reported to significantly increase the risk of new agility-related injuries [7], a finding confirmed by our study. However, in our sample, having multiple agility-related injuries significantly increased the odds of injury, whereas having only one previous injury was not a major factor. Attention should be paid to rehabilitation and conditioning of dogs after an injury, especially in dogs with a history of multiple agility-related injuries. After injury rehabilitation, additional conditioning is required to re-gain full function to meet the demands of the sport [28]. Physiotherapist-guided rehabilitation and conditioning were used for only one-fifth of the cases in our study, suggesting that there is room for improvement. Additionally, there may be certain properties of dogs, such as conformation or personality traits, that predispose them to being repeatedly injured even after appropriate rehabilitation.

Starting age has not been evaluated as a risk factor in previous studies, but it was an important factor in our model. Starting course-like training, where dogs perform obstacle sequences including jumps, at an older age doubled the odds of agility-related injury during 2019 for each year added. Traditionally, high-intensity activities have been discouraged in dogs during their first 12–18 months before all growth plates have closed [28]. Based on anecdotal data, dogs that repeatedly perform jumps or weaves at a young age have been proposed to be at increased risk for chronic injuries later during their career [28]. Our findings were not in line with this. In contrast, our results suggested that with a median starting age of 12 months for course-like training, starting early, probably before closure of all growth plates in many dogs, was protective for agility-related injury later in their career. Similarly, racehorses starting racing or training as two-year-olds have lower risk of injury than horses starting at an older age, probably because of better adaptation capacity at a younger age [29]. However, additional analysis of our sample revealed no association between agility-related injury during the whole career (2019 or earlier) and starting age. Thus, the initial finding could be coincidental, describing only the sample used for the regression models. Additionally, young dogs just starting agility did not generally meet our inclusion criteria, as only competing dogs were included and most had started training before 2019. Thus, whether early start affects the risk of injury during the early training stages could not be evaluated in this study. More research is needed to set recommendations for the training of young dogs.

Frequency of training sessions has not been related to agility-related injury in previous studies [5,7]. However, the training frequency was asked for the past year before participation in the survey, which may not represent training frequency prior to the injury. In our study, high training frequency prior to the injury was associated with higher likelihood of agility-related injury in the training frequency model. This could have been simply due to high exposure to agility in dogs that train often. However, weekly training time was not associated with injury. Dogs with high training frequency may not have had sufficient time to recover from training before the next session. In human athletes, there is conflicting evidence on the association between training frequency and injury [30]. However, an increase in the short-term amount of training in relation to the long-term amount increases the risk for injury (acute: chronic workload ratio) [30,31]. In our sample, the high training frequency prior to injury may indicate a peak in training frequency, possibly with an abrupt increase in training load in some dogs. However, we only asked about the usual training frequency during the preceding three months, not allowing for a detailed evaluation of the variation in training over the weeks. A prospective study design with repeated surveys or objective activity measurements is recommended to track training load in detail.

Competition frequency, like training frequency, has not been associated with agility-related injuries previously, but the earlier surveys did not include questions that recorded routines prior to injury [5,7]. In our study, a moderate frequency of competition runs was associated with lower odds of agility-related injury compared with dogs competing at low or high frequency. Dogs competing at a low frequency, on average less than 1.5 runs a month, may be insufficiently prepared for the demands of the sport compared with dogs competing more often, and competition runs may represent an abrupt increase in high-intensity activity for these dogs. A high average number of monthly competition runs is the result of a higher frequency of competition days, a higher number of runs per competition day, or both. As injuries often occurred during the second or later runs, a high number of daily runs might increase the risk of competition-related injury.

The presence of a lumbosacral transitional vertebra (LTV) was another major risk factor in all of our models. To our knowledge, this unexpected finding has not been reported previously. The LTV has been described to be associated with cauda equina syndrome [32]. However, in that study there were no dogs that had a separation of the first spinous process from the median crest of the sacrum (LTV1) [32], which was the most common type in our sample. Anecdotally, LTV1 is considered to be a mild abnormality, not necessarily of any clinical relevance. In dogs with LTV, including dogs with LTV1, the length of the L7 in relation to the L6 is increased compared with dogs without LTV [33]. The kinematics of the back may be affected in clinically sound dogs with radiographic changes in the lumbosacral junction; however, only a couple of dogs in that study had LTV and the grade was not specified [34]. Thus, the information on clinical and biomechanical effects of LTV, especially in dogs with a separation of the first spinous process from the median crest of the sacrum, is sparse and further research is needed. Agility could provoke clinical signs associated with abnormalities in the lumbosacral region, especially in dogs jumping higher fences, which results in greater extension of the lumbar spine [26]. Additionally, if the function of the hind limbs or back is compromised by the LTV or associated abnormalities, it could affect risk of injury of other tissues. Alternatively, owners and handlers of dogs with known LTV may observe their dogs more carefully or be more likely to rest their dog in case of minor signs.

Our study showed that participation in other physically demanding sports may protect from agility-related injury, a finding that has not been reported previously. These other sports may provide conditioning that prepares the dogs for the physical demands of agility. Similarly, human athletes highly specialized in one sport have greater risk of overuse injury than athletes with less specialization [35,36]. In humans, this effect may be independent of amount of training [35]. Low diversity in the movement patterns practiced by specialized athletes may affect the development of neuromuscular skills that protect from injury [37]. Additionally, certain parts of the body may get insufficient rest from repetitive activities [37]. Variation in physical exercise may thus be advisable also for agility dogs.

To our surprise, competition speed was not associated with injury, suggesting that other factors had greater effect on the odds of injury. Alternatively, the method of evaluating dogs’ speed may be inaccurate; competition speed is calculated in the competition results database from course time of a faultless run divided by course length measured by the judge. However, the expected route measured by the judge may differ from the actual route of the dog over the course. The course time is also affected by the handler’s ability to train obstacle skills and to guide the dog on the course. Courses differ in the number and tightness of turns as well as in obstacles, and difficulty increases with level. Thus, the competition speed value may not reliably represent a dog’s running speed in agility.

Musculoskeletal care has been associated with agility-related injury in previous studies, but the timing of the therapies in relation to injury was not evaluated in these studies so they could also be a result of reversed causality [3,7]. In our sample, dogs receiving physiotherapy every two to three months during the year preceding injury had increased odds of injury, even when controlled for previous injuries. Thus, physiotherapy appears to be associated with injury independently from previous injuries. Possibly owners choosing to provide regular physiotherapy for their dog are more likely to notice their dog’s clinical signs and/or to restrict training or exercise in case of even minor signs. The owners may also have been taught observation or palpation skills by the physiotherapist, which allowed them to recognize minor abnormalities. It is unlikely that the finding would be explained by injuries detected during physiotherapy, as in our study clinical signs had to be evident within 24 h of agility and dogs with higher odds of agility-related injury visited the physiotherapist every two to three months. It cannot be ruled out that some practices or recommendations from the physiotherapist might have increased the risk of injury.

### 4.5. Study Design and Limitations

To our knowledge, a definition of injury per se has not been provided to respondents in previous questionnaire studies [2,4,5]. The time-loss definition, defining injury as a physical complaint resulting from the sport and leading to time lost from training and competition, is used in human sports [38,39] and was chosen for our study. Because dogs do not complain about discomfort, clinical signs observed by the owner were used instead. The owner also evaluated whether the clinical signs were caused by agility. As many agility dogs can have multiple days in between agility sessions, time lost from usual physical exercise was additionally included in our definition. This allowed for minor injuries, leading to exercise being restricted for only one or two days, to be included in this study.

A relatively high number of dogs that were reported as injured had to be excluded from this study, mainly because clinical signs were not observed within 24 h of training or competing in agility. Perhaps, the criteria outlined in the initial question of agility-related injury was not understood correctly or not read thoroughly. In some cases, clinical signs, such as pain on palpation, were detected by a physiotherapist or other professional only at a later time. Thus, it may be that these signs went initially unnoticed by the owner or the handler. However, once the time between the agility session and the clinical signs increases, it becomes more likely that the clinical signs are unrelated to agility. The 24-h criterion was chosen since we expected that in most cases clinical signs develop within this time frame. It appears that respondents may intuitively define agility-related injury in different ways. We recommend that in future questionnaire studies the injury should be defined clearly to respondents with own check boxes for each criterion used.

Most of the previous survey studies on agility-related injuries have requested details of injuries during the whole career or at least over a two-year period before participation [2,4,5]. We covered in detail injuries occurring only over one year, which is likely to have improved respondents’ ability to accurately remember passed events. Despite this, for many dogs, anatomical location and type of injury were subject to being imprecise and clarifications were sought through email. In some cases, the veterinary diagnosis described in open field was not in agreement with the checked boxes for anatomical location and/or type of the injury, highlighting the issue. Many dogs did not receive veterinary care and the location and type of injury relied on the evaluation of the owner or paraprofessional. Additionally, selection bias could have affected our results; for example, injured dogs could have been overrepresented if their owner perceived the study as more important and were therefore more likely to invest time in participation than owners of non-injured dogs. The latest injury from 2019 was included in the analyses if there were multiple injuries for one dog, as several injuries could not have been analyzed independently of each other. Additionally, the respondents are likely to remember the latest injury most accurately, improving the quality of the data. This selection criterion could have resulted in a majority of injuries being from the end of the year, possibly associated with certain conditions. However, most dogs had just one injury.

Factors included in each model were mainly the same, especially the most significant ones. To select variables for development of the multivariate models, variables with *p* < 0.1 in univariate analysis were chosen. With this threshold, a high proportion of dogs had no missing values in most of the variables chosen for the development of multivariate models. With a higher threshold, such as *p* < 0.2 or <0.25 used in some previous studies [5,7], a greater number of variables would have been chosen for the model development and therefore a greater proportion of dogs would have had missing values in at least one of the variables. This would have resulted in a markedly smaller population to be used for multivariate model development. To include as many dogs as possible in the final model, we chose to exclude some variables from it and analyzed them separately in the subgroup analyses. With a larger sample, which could be achieved by an international sample, all variables could have been included in the same model. However, an international population would not allow utilizing objective data from national competition result databases. Additionally, translating the questionnaire would be required to get sufficient samples also outside countries with English as the native language, which brings issues with possibly different meaning of questions in different languages. One should also remember that these models detect only associations, not causality. Some risk factors may also be linked to some other factors, not covered by the questionnaire.

## 5. Conclusions

Agility dogs are prone to soft tissue injuries to their front limbs, with most injuries occurring during obstacle performances. Dogs with multiple previous agility-related injuries, lumbosacral transitional vertebra, later starting age in the sport, and high training frequency appear to be at greatest risk for agility-related injury. Multiple additional, less significant factors improved our models in predicting odds of agility-related injury in our sample. Reviewing obstacle regulations could aid in reducing some obstacle-related injuries.

## Figures and Tables

**Table 1:** Injury-related variables provided by the survey.

Category	Variable
Injury	Agility-related injury during 2019 (yes/no) Number of agility-related injuries during 2019 Date of the latest agility-related injury
Context of the injury	During competition or training If training: first or second half of the training session If competition: number of runs before the injury on that day During obstacle performance (yes/no) ^1^ If obstacle-associated: Which obstacle, collision (yes/no), fall (yes/no), slipping during obstacle performance (yes/no) Slipping in between obstacles (yes/no) When injury was noticed (during or after agility) Surface of the agility field
Description of the injury	Anatomical location ^2^ Type of the injury Clinical signs
Treatment	Veterinary care (yes/no) If veterinary care was sought: the time of diagnosis in relation to injury Treatments
Recovery	Recovery time to normal exercise Recovery time to performance level prior to injury

^1^ Including take-off to and landing from the obstacle. ^2^ Picture describing the following anatomical locations was provided for respondents: Head, eye, neck, back, pelvis, tail, rib cage, scapula, shoulder, brachium, elbow, antebrachium, carpus, metacarpal region, toe or nail (front limb), paw pads (front limb), thigh, groin, stifle, crus/shank, hock, metatarsal region, toe or nail (hind limb), and paw pads (hind limb).

**Table 2:** Number of completed runs prior to competition-related injury (*n* = 34).

Number of Runs	Number of Dogs	Proportion of Dogs with Competition-Related Injury
0	8/34	23.5%
1	16/34	47.1%
2	5/34	14.7%
3	5/34	14.7%

**Table 3:** Obstacles involved in injuries of 63 dogs. Performance of each obstacle is presented in Video S1.

Obstacle	Number of Dogs	Proportion of Dogs with Obstacle-Related Injury
Bar jump	23/63	36.5%
Dogwalk	11/63	17.5%
A-frame	10/63	15.9%
Open tunnel	10/63	15.9%
Weave poles	4/63	6.3%
Seesaw	2/63	3.2%
Spread jump	1/63	1.6%
Tire	1/63	1.6%
Long jump	1/63	1.6%

**Table 4:** Field surfaces at the time of injury (*n* = 111).

Field Surface	Number of Dogs	Proportion of Dogs
Artificial turf with rubber filling	39/111	35.1%
Dirt or sand	25/111	22.5%
Artificial turf without filling	21/111	18.9%
Artificial turf with cork filling	14/111	12.6%
Artificial turf with sand filling	6/111	5.4%
Natural grass	5/111	4.5%
Rubber mat	1/111	0.9%

**Table 5:** Most commonly injured anatomical locations (*n* = 119).

Anatomical Location	Number of Dogs	Proportion of Dogs
Back	23/119	19.3%
Brachium	19/119	16.0%
Scapular region	16/119	13.4%
Shoulder	15/119	12.6%
Pelvis	14/119	11.8%
Thigh	13/119	10.9%
Ribcage	12/119	10.1%
Digit (front limb)	12/119	10.1%
Carpus	11/119	9.2%
Nail of a front limb	11/119	9.2%
Neck	10/119	8.4%

No injuries to eye, paw pads of hind limbs, or abdominal region were reported.

**Table 6:** Therapies and paraprofessionals used in treatment of agility-related injuries (*n* = 118).

Treatment	Number of Dogs	Proportion of Dogs
Exercise restriction	91/118	77.1%
Medical treatment	55/118	46.6%
Physiotherapy	50/118	42.4%
Rehabilitation/conditioning as part of physiotherapy	25/118	21.2%
Osteopathy	15/118	12.7%
Massage	14/118	11.9%
Laser therapy	13/118	11.0%
Wound care	11/118	9.3%
Other therapies ^1^	9/118	7.6%
Taping	8/118	6.8%
Acupuncture	8/118	6.8%
Surgery	6/118	5.1%
Craniosacral therapy	5/118	4.2%
Splint or cast	4/118	3.4%
Cryotherapy	4/118	3.4%

^1^ Other therapies included, for example, myofascial therapy (*n* = 3) and magnet therapy (*n* = 1).

**Table 7:** Severity of the injury graded by recovery time to previous level in agility (*n* = 99).

Severity	Number of Dogs	Proportion of Dogs
Minor (<3 weeks)	26/99	26.3%
Moderate (3 to <8 weeks)	29/99	29.3%
Severe (≥8 weeks, with return to agility)	33/99	33.3%
Career ending	11/99	11.1%

**Table 8:** Elements of a usual warm-up and cool-down.

Item	Warm-Up (*n* = 118)	Cool-Down (*n* = 118)
Exercising on leash	93.2% (*n* = 110/118)	92.4% (109/118)
Exercising off leash	61.0% (*n* = 72/118)	55.9% (66/118)
Walking	61.9% (*n* = 73/118)	70.3% (83/118)
Running	72.9% (*n* = 86/118)	50.8% (60/118)
Sprinting	35.6% (*n* = 42/118)	1.7% (2/118)
Tricks	69.5% (*n* = 82/118)	4.2% (5/118)
Tug play	31.4% (*n* = 37/118)	2.5% (3/118)
Active stretches	31.4% (*n* = 37/118)	4.2% (5/118)
Passive stretches	13.6% (*n* = 16/118)	10.2% (12/118)
Habituation to the field surface	32.2% (*n* = 38/118)	Not applicable
Obstacle performances as part of warm-up	20.3% (*n* = 24/118)	Not applicable
Other ^1^	1.7% (*n* = 2/118)	3.4% (4/118)

^1^ Other elements included, for example, playing with other dogs, massage, or swimming.

**Table 9:** Distribution of treatment frequency of 118 dogs by massage therapist, physiotherapist, osteopath, or other professional during the year preceding injury.

Professional	At Least Once a Month	Every Two to Three Months	Less Often	Not at All
Massage therapist	16.1% (*n* = 19/118)	30.5% (*n* = 36/118)	19.5% (*n* = 23/118)	33.9% (*n* = 40/118)
Physiotherapist	6.8% (*n* = 8/118)	31.4% (*n* = 37/118)	27.1% (*n* = 32/118)	34.7% (*n* = 41/118)
Osteopath	1.7% (*n* = 2/118)	14.4% (*n* = 17/118)	20.3% (*n* = 24/118)	63.6% (*n* = 75/118)
Other	1.7% (*n* = 2/118)	16.1% (*n* = 19/118)	9.3% (*n* = 11/118)	72.9% (*n* = 86/118)

**Table 10:** Musculoskeletal diagnoses unrelated to the agility-related injury of 2019.

Diagnosis	Number of Dogs	Proportion of Dogs
Lumbosacral transitional vertebra	26/119	21.8%
Hip dysplasia	16/119	13.4%
Other muscle injury	5/116	4.3%
Fracture	5/119	4.2%
Patellar luxation	4/119	3.4%
Disease of the elbow	3/119	2.5%
Carpal sprain	2/118	1.7%
Sprain of toe	2/118	1.7%
Injury of biceps tendon or muscle	2/118	1.7%
Other tendon injury	2/118	1.7%
Osteochondrosis/osteochondritis dissecans	2/119	1.7%
Injury of supraspinatus muscle or tendon	1/118	0.8%
Shoulder instability/medial shoulder syndrome	1/119	0.8%

## Data Availability

The data presented in this study are available on request from the corresponding author.

## Supplementary Materials

The following supporting information can be downloaded at: https://www.mdpi.com/article/10.3390/ani12030227/s1, File S1: Link to the final questionnaire, Table S1: Breeds of dogs with agility-related injury during 2019, Table S2: Variables associated with increased or decreased odds of agility-related injury during 2019 in univariate logistic regression analysis. Video S1: Demonstration of agility obstacles.
