# Supplementary material for: Part II of Finnish Agility Dog Survey: Agility-Related Injuries and Risk Factors for Injury in Competition-Level Agility Dogs
Source: Animals (Basel). 2022 Jan 18;12(3):227. doi: 10.3390/ani12030227 (PMC8833498; doi:10.3390/ani12030227)
Supplement: Supplementary file 1 [file animals-12-00227-s001.zip › S1_TableS1_TableS2.pdf]

animals

Supplement S1. Link to the final questionnaire: https://elomake.helsinki.fi/lomakkeet/105548/lomake.html

Table S1. Breeds of dogs with agility-related injury during 2019.

Breed	Proportion of dogs (n=119)
Border Collie	23.5%
Shetland Sheepdog	10.9%
Belgian Shepherd	7.6%
Collie	5.9%
Australian Kelpie	5.0%
Spanish Water Dog	5.0%
Australian Shepherd	4.2%
Kooikerhondje	2.5%
Mudi	2.5%
Parson Russell Terrier	2.5%
Dachshund	1.7%
German Pinscher	1.7%
Medium-sized Poodle	1.7%
Mixed Breed	1.7%
Nova Scotia Duck Tolling Retriever	1.7%
Pyrenean Sheepdog	1.7%
Standard Poodle	1.7%
Alaskan Malamute	0.8%
Cavalier King Charles Spaniel	0.8%
Chihuahua	0.8%
Danish-Swedish Farmdog	0.8%
Finnish Lapponian Dog	0.8%
German Shepherd	0.8%
Icelandic Sheepdog	0.8%
Irish Soft Coated Wheaten Terrier	0.8%
Jack Russell Terrier	0.8%
Lancashire Heeler	0.8%
Lapponian Herder	0.8%
Löwchen	0.8%
Medium Spitz	0.8%
Miniature Pinscher	0.8%
Miniature Poodle	0.8%
Norfolk Terrier	0.8%
Phaléne	0.8%
Portuguese Podengo	0.8%
Puli	0.8%
Pumi	0.8%
Standard Schnauzer	0.8%
Swedish Vallhund	0.8%

Table S2. Variables associated with increased or decreased odds of agility-related injury during 2019 in univariate logistic regression analysis.

Variable	Injury (n=119)	No injury (n=745)	OR (95% CI)	p-value
Age[1]	119	745	1.084 (0.998–1.177)	0.056
Sex				0.035
Male	59	293	Reference	
Female	60	452	0.659 (0.447–0.972)	0.035
Height[2]	119	745	1.032 (1.012–1.054)	0.002
Weight[3]	119	745	1.040 (1.011–1.070)	0.006
Weight / height ratio[4]	119	745	12.356 (1.519–100.535)	0.019
Height category				0.064
Large	42	197	Reference	
Small Large	31	162	0.898 (0.540–1.492)	0.677
Medium	24	180	0.625 (0.364–1.074)	0.089
Small	13	151	0.404 (0.209–0.779)	0.007
Extra Small	9	55	0.768 (0.352–1.674)	0.506
Breed				0.048
Non-Border Collie	91	625	Reference	
Border Collie	28	120	1.603 (1.005–2.555)	0.048
Previous agility-related injury (yes/no)	66	159	4.843 (3.209–7.307)	<0.001
Number of previous agility-related injuries				<0.001
0	560	48	Reference	
1	107	12	1.308 (0.673–2.546)	0.429
≥2	52	48	10.769 (6.593–17.591)	<0.001
Non-agility-related musculoskeletal injury (yes/no)	45	187	1.918 (1.271–2.895)	0.002
Diagnosis of lumbosacral transitional vertebra	26	89	2.061 (1.265–3.357)	0.004
Sprain of toe prior to 2019	2	2	6.362 (0.887–45.607)	0.066
Carpal sprain prior to 2019	2	2	6.371 (0.889–45.669)	0.065
Dog's competition years in agility[1]	119	741	1.078 (0.989–1.176)	0.089
Age at which course-like training was started[1]	115	735	1.304 (1.033–1.646)	0.026
Age at which jumps were set at competition height[1]	118	744	1.355 (1.069–1.719)	0.012
Number of competition runs per month[5]				<0.001
<1.5 runs / month	42	259	Reference	
1.5 to <3.0 runs / month	16	221	0.446 (0.244–0.816)	0.009
≥3.0 runs / month	61	265	1.419 (0.925–2.179)	0.109
Competition speed[6]	104	669	1.457 (1.023–2.075)	0.037
Proportion of clean runs[7]	119	745	0.278 (0.068–1.145)	0.076
Frequency of visits to physiotherapist[8]				0.011
Not at all	41	357	Reference	
At least once a month	8	36	1.935 (0.843–4.444)	0.120
Every two to three months	37	145	2.222 (1.369–3.607)	0.001
Less often	32	207	1.346 (0.822–2.204)	0.237
Frequency of visits to other professionals for musculoskeletal care[8]				0.001
Not at all	86	624	Reference	
At least once a month	2	30	0.484 (0.114–2.060)	0.326
Every two to three months	19	49	2.813 (1.582–5.003)	<0.001

Less often	11	42	1.900 (0.943–3.831)	0.073
Passive stretches as part of usual cool-down[5]	12	39	2.046 (1.038–4.033)	0.039
Participation in other physically demanding activities[5]	15	180	0.457 (0.259–0.806)	0.007
Performance technique on A-frame				0.012
Stopped contact	39	181	Reference	
Running contact	65	472	0.639 (0.415–0.985)	0.042
Other or in between	5	86	0.270 (0.103–0.709)	0.008
Frequency of training sessions[5]				0.002
<2 sessions / week	51	309	Reference	
2 sessions / week	49	213	1.394 (0.907–2.141)	0.129
>2 sessions / week	18	34	3.208 (1.685–6.105)	<0.001
Main surface in training and competitions[5]				0.050
Artificial turf with rubber filling	38	200	Reference	
Artificial turf without filling	25	88	1.495 (0.851–2.627)	0.162
Artificial turf with cork filling	14	64	1.151 (0.587–2.260)	0.682
Dirt or sand	31	81	2.014 (1.174–3.457)	0.011
Other (e.g. artificial turf with sand filling, natural grass, rubber mat, fibre-sand mix, or horse-riding surface)	9	18	2.632 (1.100–6.294)	0.030
Field surface at time of injury[9]				0.040
Artificial turf with rubber filling	39	200	Reference	
Artificial turf without filling	21	88	1.224 (0.681–2.201)	0.500
Artificial turf with cork filling	14	64	1.122 (0.573–2.198)	0.738
Dirt or sand	25	81	1.583 (0.900–2.784)	0.111
Other (e.g. artificial turf with sand filling, natural grass, rubber mat, fibre-sand mix, or horse-riding surface)	12	18	3.419 (1.525–7.662)	0.003

[1] For one year increase, the odds increase by OR.
[2] For one centimetre increase, the odds increase by OR.
[3] For one kilogram increase, the odds increase by OR.
[4] For one unit increase in kg/cm ratio, the odds increase by OR.
[5] Routines during the three-month period preceding the injury in injured dogs and during 2019 in non-injured dogs.
[6] For one unit increase in m/s, the odds increase by OR.
[7] For one percentage increase, the odds increase by OR.
[8] Routines during the one-year period preceding the injury in injured dogs and during 2019 in non-injured dogs.
[9] Main surface during training and competition was used for non-injured dogs.
